# NR0B1 Gene Variants as Rare Forms of Primary Adrenal Insufficiency in Children: Case Report and Narrative Review

**DOI:** 10.3390/genes17060640

**Published:** 2026-05-31

**Authors:** Ilaria Montafia, Sotirios Dimarakis, Cristina Partenope, Ivana Rabbone, Simonetta Bellone, Antonella Petri, Simona Mellone, Mara Giordano, Flavia Prodam

**Affiliations:** 1 Department of Health Sciences, University of Piemonte Orientale “Amedeo Avogadro”, 28100 Novara, Italy; montafiai@gmail.com (I.M.); sotiridimar92@gmail.com (S.D.); ivana.rabbone@uniupo.it (I.R.); simonetta.bellone@med.uniupo.it (S.B.); simona.mellone@uniupo.it (S.M.); mara.giordano@med.uniupo.it (M.G.); 2 Department of Pediatrics, AOU Maggiore della Carità, 28100 Novara, Italy; antonella.petri@maggioreosp.novara.it; 3 Laboratory of Genetics, Struttura Complessa a Direzione Universitaria (SCDU) Biochimica Clinica, Ospedale Maggiore della Carità, 28100 Novara, Italy; 4 Department of Translational Medicine, University of Piemonte Orientale “Amedeo Avogadro”, 28100 Novara, Italy; flavia.prodam@med.uniupo.it

**Keywords:** primary adrenal insufficiency, children, NR0B1, exome sequencing, X-linked adrenal hypoplasia congenita

## Abstract

Primary adrenal insufficiency (PAI) is a severe and potentially life-threatening condition characterised by the inability of the adrenal cortex to produce enough glucocorticoids and/or mineralocorticoids. The clinical signs of PAI are primarily due to deficient steroid hormone synthesis and include weight loss, orthostatic hypotension secondary to dehydration, hyponatremia, hyperkalaemia, and hypoglycaemia. In the paediatric population, PAI is most commonly associated with inherited monogenic disorders, particularly enzyme deficiencies. X-linked adrenal hypoplasia congenita (AHC) is a rare condition caused by deletions or single-nucleotide variants in the NR0B1 (DAX1) gene, which encodes the DAX1 protein expressed in the adrenal cortex, gonads, hypothalamus and pituitary gland. Although molecular genetics has significantly expanded our understanding of the aetiology of PAI, clinical diagnosis remains challenging when the initial hormonal findings are atypical, often delaying recognition and treatment. Pathogenic variants of DAX1 can lead to a spectrum of phenotypes, ranging from isolated adrenal insufficiency (AI) to complex syndromic presentations combining AI with hypogonadotropic hypogonadism and impaired spermatogenesis. Here, we report a case of a male patient with AI due to a de novo pathogenic variant in the NR0B1 gene. Furthermore, we provide a non-systematic review of the available literature on the diagnostic challenges facing and clinical variability in AHC, with a particular focus on the paediatric population. This case highlights the importance of a stepwise, comprehensive diagnostic approach to suspected PAI, particularly when initial biochemical and genetic testing is inconclusive. Considering rare causes—such as NR0B1 pathogenic variants in men—can be crucial for establishing a definitive diagnosis, with significant implications for the management of patients and their families.

## 1. Introduction

Primary adrenal insufficiency in children includes a heterogeneous group of rare disorders that may result from impaired adrenal development, defects in steroidogenesis, adrenal destruction, metabolic diseases, or syndromic/genetic conditions. Congenital adrenal hyperplasia due to 21-hydroxylase deficiency remains the most frequent cause, but adrenal hypoplasia congenita, metabolic disorders, autoimmune adrenalitis, and other monogenic conditions should also be considered, particularly in patients with atypical biochemical or clinical features [1]. A stepwise diagnostic approach integrating hormonal evaluation and genetic testing is therefore essential for accurate diagnosis and management. Variants of the NR0B1 gene, also known as DAX1, underlie a condition named X-linked adrenal hypoplasia congenita (OMIM 300200, also known as AHC to distinguish it from CAH) [2], a rare but clinically significant cause of PAI. The gene, located at Xp21.3–p21.2, was first described in 1994 [3] and encodes a nuclear receptor involved in several endocrine pathways, playing a pivotal role in the development and maintenance of the adrenal cortex, hypothalamus, anterior pituitary, and gonads [4,5,6,7].

The clinical expression of AHC is heterogeneous. The majority of affected males present with PAI during infancy or early childhood, frequently culminating in acute adrenal crisis, characterised by hypotension, hypoglycaemia, electrolyte imbalance, and potential cardiovascular collapse. Nevertheless, increasing numbers of reports document delayed or adult-onset forms. Such atypical cases can be easily overlooked or misdiagnosed, as they may mimic other aetiologies. A cardinal feature across the spectrum is hypogonadotropic hypogonadism (HH), which manifests as incomplete or absent pubertal development, reduced testicular volume, and infertility.

Classical, early-onset AHC typically results from complete loss-of-function alleles, whereas milder or late-onset presentations are more frequently associated with missense or frameshift variants retaining partial protein activity. These observations suggest that AHC exists on a continuum of phenotypic expression, likely modulated by residual protein function, modifier genes, and environmental factors [8,9].

We report a case of a child with a DAX1 variant and conducted a non-systematic review of the available literature on the disease, selecting clinically relevant reports and emphasising the importance of genetic testing for precise diagnosis.

## 2. Methods

DNA was extracted from 200 μL of whole blood (anti-coagulated with EDTA) using ReliaPrepTM Blood gDNA Miniprep System (Promega, Fitchburg, WI, USA). Library preparation was performed using a Magnis SureSelect XT HS2 NGS Target Enrichment Kit for Illumina multiplexed sequencing in combination with a Magnis SureSelect Custom Constitutional Panel 17 Mb (Agilent, Santa Clara, CA, USA), according to the manufacturer’s instructions. The sequencing panel targeted all coding exons and flanking exon–intron splice junction regions of 5227 clinically relevant genes. The enriched libraries were sequenced using 150 bp paired-end reads on an Illumina NextSeq1000 platform (Illumina, San Diego, CA, USA). The average sequencing depth was 179×, with 97.04% of target bases covered at ≥20×, 90.37% at ≥50×, and 73.40% at ≥100×. Sequencing reads passing quality control filters were aligned to the human reference genome build GRCh37/hg19, and variant calling was performed using enGenome eVai software (v3.9; evai.engenome.com (accessed on 7 March 2026). Data analysis was subsequently carried out using a customised bioinformatics pipeline. Only variants with a minor allele frequency (MAF) < 1% in public databases (1000 Genomes Project, ExAC, and gnomAD) were considered, while synonymous variants were excluded from further analysis. Among the filtered variants, priority was given to frameshift, nonsense, splice-site, and missense variants predicted to be pathogenic by at least four in silico prediction tools (SIFT, PolyPhen-2, MutationTaster, and CADD). Variant pathogenicity was assessed according to the consensus guidelines of the American College of Medical Genetics and Genomics (ACMG) and the Association for Molecular Pathology (AMP). The analysis did not allow exclusion of the presence of copy number variants (CNVs) in genes associated with AHC. The variant identified by clinical exome sequencing was validated by Sanger sequencing using a BigDye Terminator v1.1 Cycle Sequencing Kit (Applied Biosystems, Foster City, CA, USA) and analysed on a SeqStudio Genetic Analyzer (Applied Biosystems).

## 3. Case Description

A male newborn was referred to the Paediatric Endocrine Centre for clinical signs of adrenal crisis and salt wasting. Pregnancy was uneventful except for low levels of estriol (1.23 ng/mL); amniocentesis was then performed, foetal karyotype was 46 XY, and Xp22.3 deletion was excluded. Placental steroid sulfatase deficiency was suspected, and C-section was planned at full term. At birth the APGAR score was 9/9. The baby’s birth weight and length were adequate. His examination was unremarkable; his external male genitalia were normal. No complications occurred during the perinatal period. However, at 10 days of life, the baby was admitted to the Neonatal Intensive Care Unit for failure to thrive, vomiting, and breastfeeding difficulties. His blood tests showed hyponatraemia and hyperkalaemia, with a markedly elevated serum potassium level of 8.2 mEq/L. Haemolysis was not reported, and no documentation of electrocardiographic changes was available. Emergency management included the initiation of intravenous maintenance fluids, which corrected the hyponatraemia but did not fully normalise the hyperkalaemia. Oral calcium gluconate was subsequently administered, leading to partial improvement in the serum potassium levels. After paediatric endocrine consultation, hormonal tests were performed (Table 1), showing borderline levels of cortisol, low levels of 17α-hydroxyprogesterone (17OHP) and dehydroepiandrosterone sulphate (DHEAS), and elevated ACTH, testosterone and androstenedione. Renin levels were very high as well, but blood pressure, blood gas analysis and urine output were normal. Age-specific reference values are reported in Supplementary Table S1. Neonatal brain and abdomen ultrasound did not show any abnormalities. He was diagnosed with PAI and started on oral sodium chloride supplementation and hormonal replacement therapy (hydrocortisone 20 mg/m^2^/day and fludrocortisone 0.1 mg/day). Electrolyte balance, weight and clinical conditions gradually improved.

Although hormonal results were atypical for CAH, we performed chromosomal microarray analysis (CMA) and gene sequencing for CYP21A2, CYP11B1, HSD3B2 and CYP17A1 genes, which were all normal. The next-generation sequencing panel also ruled out pseudohypoaldosteronism type I.

**Figure 1:**
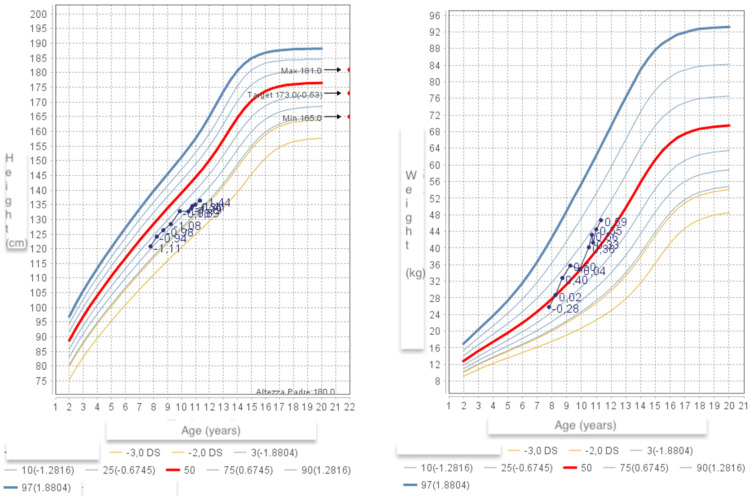
Height (**left**) and weight (**right**) growth charts of the patient.

During follow-up he did not present with adrenal crisis and grew regularly in height and weight (Figure 1), with bone age always matching his chronological age. For apparent testicular enlargement, at three years of age, he underwent a GnRH stimulation test, which showed prepubertal gonadotropins (LH peak 0.2 mIU/mL, FSH peak 1.6 mIU/mL), but elevated testosterone levels (21.5 ng/dL); tumour markers were negative, and brain MRI was normal. Repeated testicular and adrenal ultrasounds were normal.

Sodium chloride supplementation was discontinued at 1 year of age, and hydrocortisone treatment was continued at a dosage of approximately 15 mg/m^2^ per day, divided into four daily doses to optimise coverage and biochemical control; therapy was titrated based on blood tests and growth. He maintained normal electrolyte balance throughout the years, but ACTH values were persistently very high with 17OHP, DHEAS and testosterone at the lower limit of normal (Table 1).

**Figure 2:**
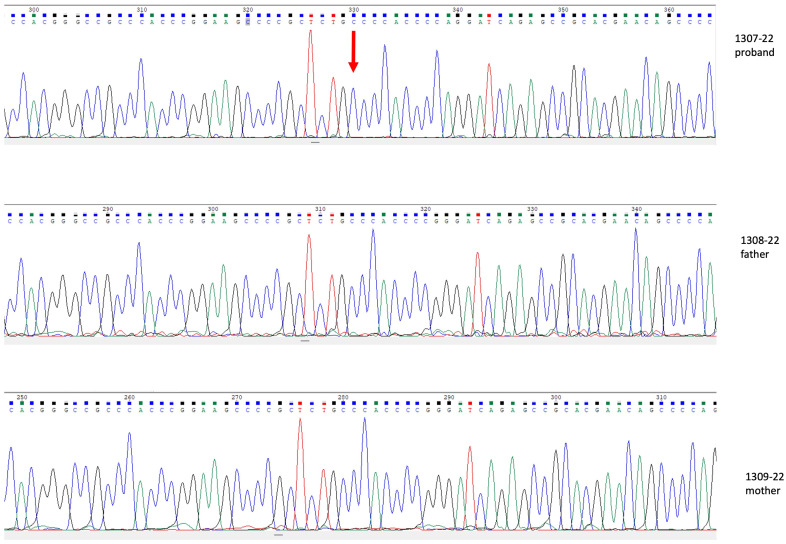
A representative image of the read alignment showing the insertion of a cytosine in exon 1 of the NR0B1 gene (NM_000475.5:c.347dup; p.Arg117GlnfsTer21), shown with a red arrow in the first alignment. The variant was confirmed by Sanger sequencing. Segregation analysis revealed that the variant arose de novo, being absent in the parents (second and third alignments).

To pursue a definitive diagnosis, we performed exome sequencing and found a novel hemizygous X-linked variant c.347dup (p.Arg117GlnfsTer21) in NR0B1 (DAX1) gene. This insertion causes a frameshift, leading to the creation of a premature stop codon 21 amino acids downstream. The variant is absent from population databases, including ExAC, dbSNP, ESP, gnomAD, ClinVar, and the 1000 Genomes Project, and has been classified as pathogenic according to the ACMG guidelines (criteria: PVS1_Strong, PS2, PM2_Supporting) and is consistent with the clinical diagnosis of AHC. Sanger sequencing analysis of the parents confirmed that the variant arose de novo (Figure 2).

**Figure 3:**
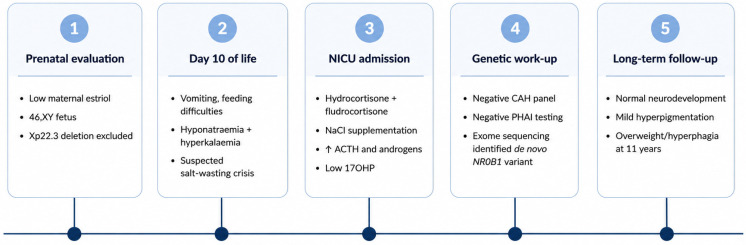
Chronological overview of patient’s diagnostic work-up, treatment, and endocrine follow-up.

At the last evaluation the patient was 11 years old, prepubertal, in good clinical conditions and with adequate cognitive and motor development. His skin was slightly darker, in agreement with ACTH levels. His growth in height was regular, but his weight progressively increased, and he was overweight at the last visit (BMI 22.8 Kg/m^2^; SDS 1.16), reporting hyperphagia, assessed through the Dykens questionnaire [10] (total score 22). Dietary advice was given to the family. We managed to achieve better hormonal control with hydrocortisone granules rather than tablets at the same dosage (13.5 mg/m^2^/day) and continued fludrocortisone treatment (0.15 mg/day). ACTH levels progressively decreased. The patient’s clinical course and major diagnostic, therapeutic, and follow-up milestones are summarised in Figure 3.

## 4. Discussion

Adrenal hypoplasia congenita (AHC) due to pathogenic variants in NR0B1 (DAX1) is a rare but important cause of primary adrenal insufficiency in male patients. The disorder is characterised by marked clinical heterogeneity, ranging from severe neonatal adrenal crisis to delayed-onset forms diagnosed later in life. Early recognition is crucial because affected patients require lifelong glucocorticoid and mineralocorticoid replacement and careful monitoring of pubertal development and fertility.

Our patient presented during the neonatal period with salt-wasting adrenal insufficiency, requiring prompt hormonal replacement therapy. However, the biochemical profile was atypical for congenital adrenal hyperplasia, with low 17OHP levels despite markedly elevated ACTH concentrations. Initial targeted genetic investigations for more common causes of adrenal insufficiency were inconclusive, and only subsequent exome sequencing identified a novel de novo hemizygous pathogenic variant of NR0B1 (c.347dup; p.Arg117GlnfsTer21), confirming the diagnosis of AHC. The identified variant consists of a single-nucleotide duplication at coding position 347, leading to a frameshift starting at codon 117. This alteration changes the downstream amino acid sequence and introduces a premature termination codon (PTC) 21 codons downstream. Given the early location of the PTC within exon 1 of NR0B1, the variant is predicted to cause a loss of function, either through the production of a truncated, non-functional protein or through the degradation of the mutant transcript via nonsense-mediated mRNA decay (NMD) [11].

According to The Human Gene Mutation Database (www.hgmd.cf.ac.uk, accessed on 7 March 2026), more than 200 pathogenic variants of NR0B1 are known, together with deletions of exons or of the entire gene, most of which are located at the carboxyl terminal of the protein and are nonsense or frameshift missense variants. Loss-of-function variants of NR0B1 are common pathogenic mechanisms [12]. In another case report of a patient with a variant of NR0B1 gene [13], genetic testing detected a hemizygous variant at position 323 of exon 1 (c.323C>A p. (Ser108*)), with the generation of a truncated protein.

Currently, no validated protocols exist that allow the precise early neonatal diagnosis of AHC. In selected situations, prenatal suspicion could be possible, and knowledge of an affected family member could guide targeted surveillance at birth. Additionally, maternal estriol levels during pregnancy may provide indirect evidence of impaired foetal adrenal steroidogenesis. Low estriol levels can occur in several disorders of steroid biosynthesis; however, in a male foetus, when more common causes have been excluded and there is a positive family history of AHC, this biochemical finding may hold significant diagnostic value [14,15]. In our case report, low maternal estriol levels were detected during routine prenatal screenings. As supported by the literature, this finding may indicate a steroidogenesis disorder. For this reason, genetic testing for a deletion at Xp22.3—including the steroid sulphatase gene—was performed on amniocentesis and was negative.

Moreover, adrenal insufficiency caused by a pathogenic DAX1 variant is also associated with HH, which often emerges later in childhood or adolescence and manifests with varying degrees of severity [5]. Reduction in testosterone and inhibin B may be observed even in the pre-pubertal period. In these cases, it is very important to closely monitor the pubertal status, with a plan to commence testosterone replacement if spontaneous puberty fails to occur, since exogenous gonadotropins seem to fail to stimulate complete pubertal development [16]. DAX1-related damage seems to profoundly impair Sertoli cell function and, consequently, spermatogenesis. Although early reports, such as that by Seminara et al. [17], suggest that fertility in patients with AHC is highly unlikely, more recent findings challenge this assumption. Wang et al. [8] described 4 out of 14 patients with adult-onset AHC who were able to achieve fatherhood via spontaneous conception or successful assisted reproduction. These observations suggest that the late-onset forms of AHC are associated with the better preservation of gonadal function compared to early-onset cases [18].

Although delayed puberty is more common, few patients with AHC present with transient manifestations of gonadotropin-independent or -dependent precocious puberty or ACTH-dependent precocious puberty. The described patient provided a complex picture: low 17-OHP and DHEAS with elevated ACTH (compatible with AHC) but also elevated neonatal testosterone and androstenedione, followed by a transient testosterone rise at age 3. The GnRH test confirmed a transient, gonadotropin-independent rise in testosterone levels, with a subsequent spontaneous reduction in the following months. This presentation suggests the involvement of several pathophysiologic mechanisms that cannot be explained by classic adrenal insufficiency solely. Physiological mini-puberty in male infants produces testosterone peaks at approximately 1 month of age, with levels declining to prepubertal values by 7 months. The neonatal testosterone elevation of the patient could be due to this phenomenon; however, mini-puberty cannot explain the transient testosterone rise at three years of age [19,20].

ACTH-driven Leydig cell steroidogenesis is well-documented in AHC [21,22]. Chronic ACTH excess due to primary adrenal insufficiency can directly stimulate testicular Leydig cells via melanocortin-2 receptors (MC2Rs), which are highly expressed in foetal and neonatal testes.

As reported by Nagel et al., spontaneous activation of Leydig cells has been documented, apparently independent of ACTH stimulation (although elevated ACTH levels were suspected to trigger testosterone production), occurring simultaneously with suppressed adrenal androgen production [23]. The authors emphasised the need for high doses of hydrocortisone (20 mg/m^2^/day), noting that hydrocortisone alone may be insufficient to halt pubertal progression, and additional treatment may be needed. Normally, DAX1 functions as a transcriptional repressor of steroidogenesis. In boys with NR0B1 variants, loss of this repression may lead to the overexpression of testicular steroidogenic activators, thereby accounting for the autonomous or semi-autonomous activation of Leydig cells and the transient testosterone production independent of both LH and ACTH observed in these patients. So, the transient testosterone rise at age 3 with prepubertal gonadotropins is more consistent with this mechanism, considering the high ACTH levels during this period [24].

The accurate assessment of testosterone in this age group also requires the careful consideration of assay limitations. Immunoassays demonstrate poor specificity at the low concentrations typical in neonates and young children, with coefficients of variation up to 95.8% in paediatric samples. Cross-reactivity with other steroids, including DHEA, androstenedione, and their metabolites, can lead to falsely elevated results. Consistently, the Endocrine Society CAH guidelines report that approximately 40% of samples positive on immunoassay screening show normal steroid levels when measured by LC-MS/MS, which is therefore strongly recommended for precise quantification [25,26].

It is also essential to consider cannot-miss diagnoses in the evaluation of androgen excess, particularly when testosterone elevations appear transient or autonomous. Leydig cell tumours typically present between ages 5 and 10 years as a painless testicular mass or signs of precocious puberty, while adrenocortical carcinoma may manifest with DHEAS levels exceeding 20 times the upper limit of normal [27,28]. Testicular ultrasound should assess for masses, asymmetry, or microlithiasis, and adrenal imaging (CT or MRI) is indicated in cases of markedly elevated or progressive androgen levels [29].

As already mentioned, the clinical presentation is highly variable from infancy through adulthood (Table 2), and genotype–phenotype correlations remain largely unpredictable. Hyperpigmentation, which results from increased proopiomelanocortin production, may be evident, as in our patient, but it is not always present [30]. In most cases—aside from those with neonatal onset—the condition may go undetected or be misdiagnosed for years. Occasionally, aldosterone deficiency may precede cortisol deficiency at onset, such as in the case reported by Iughetti et al. [31].

Regarding statural growth, serum growth hormone (GH) and IGF1 concentrations are usually preserved in patients with pathogenic NR0B1 variants, and GH replacement therapy is therefore not routinely indicated [32]. Nevertheless, in those with concomitant GH deficiency, GH substitution may be required [33]. Our patient was growing regularly, and his IGF1 levels were in range; he was still prepubertal, and we will closely monitor his pubertal development and height. Interestingly, he recently developed overweight, with reported increased dietary intake and reduced satiety. To objectively assess hyperphagia, we administered the Dykens Hyperphagia Questionnaire. It is important to acknowledge that this questionnaire was originally developed and validated for Prader–Willi syndrome, and its application to our patient with AHC falls outside the population for which it was validated. Although recent studies have explored its applicability in other genetic conditions associated with obesity, such as leptin receptor deficiency and MC4R variants, its use in patients with AHC should be interpreted with caution and requires confirmation in future studies to evaluate reproducibility [34]. The current literature is limited regarding weight growth trajectories in children with AHC. The evidence from the congenital adrenal hyperplasia (CAH) literature shows that adult and paediatric patients exhibit a higher prevalence of overweight and obesity, with contributory factors including glucocorticoid therapy, chronological age, and parental obesity. Approximately 50% of paediatric patients with CAH are overweight, and 16–25% are obese. However, in AHC specifically, the link between adrenal insufficiency, hydrocortisone exposure, appetite regulation, and obesity remains speculative. Therefore, extrapolating findings from CAH to AHC should be framed as indirect, hypothesis-generating evidence that also requires confirmation through dedicated prospective studies [25,35,36,37,38,39]. These alterations may reflect differences in long-term adrenal hypofunction and may further enhance androgen production and decrease the therapeutic efficacy of glucocorticoids.

Given the chronic nature of primary adrenal insufficiency, this underscores the importance of long-term multidisciplinary management and structured surveillance, focusing on both adrenal function and the anticipated hypogonadotropic hypogonadism.

Ongoing monitoring of adrenal function is essential for patients requiring both glucocorticoid and mineralocorticoid replacement. This involves regular assessment of sodium, potassium, aldosterone, and plasma renin activity to evaluate mineralocorticoid status. Basal ACTH and cortisol measurements serve as important indicators of glucocorticoid sufficiency. Clinical vigilance is particularly necessary during periods of salt restriction, fluid loss from vomiting or diarrhoea, or exposure to extreme heat, as mineralocorticoid sensitivity can change with age. Any concerning signs such as postural hypotension, dizziness, fatigue, hypoglycaemia, poor weight gain, or hyperpigmentation should prompt urgent investigation, including measurement of ACTH levels and potentially a cosyntropin stimulation test [30].

Pubertal surveillance is a critical component for all patients with X-linked AHC. Patients with complete deletions of the DAX1 (NR0B1) gene often present with early-onset adrenal insufficiency and have a higher likelihood of complete hypogonadotropic hypogonadism, rarely progressing to spontaneous puberty. Even in patients who initiate spontaneous puberty, arrest is common, warranting annual monitoring of LH, FSH, and testosterone levels. A subset of patients may develop gonadotropin-independent precocious puberty, requiring careful assessment to differentiate autonomous gonadal activation from adrenal androgen excess.

**Figure 4:**
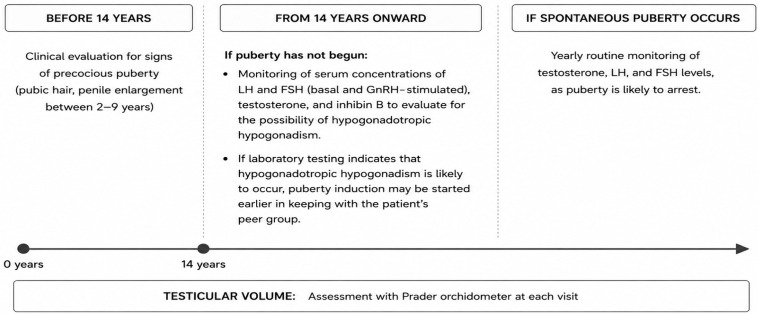
Proposed algorithm for pubertal surveillance in AHC.

Before the age of 14, clinical evaluation should focus on the early signs of puberty, such as pubic hair and penile enlargement. From age 14 onward, if puberty has not begun, laboratory assessment of LH, FSH, testosterone, and inhibin B helps identify hypogonadotropic hypogonadism and guides the timing of puberty induction, which can be tailored to align with peers. In cases of spontaneous puberty, yearly monitoring of hormone levels is recommended, along with the assessment of testicular volume at each visit using a Prader orchidometer. Notably, there is no clear genotype–phenotype correlation for predicting pubertal outcomes, and intrafamilial variability has been observed, making individualised surveillance essential for all patients with NR0B1 pathogenic variants [23,30,40]. A proposed algorithm for pubertal surveillance is summarised in Figure 4.

Family education and counselling are fundamental, addressing the high likelihood of absent or incomplete puberty and discussing potential testosterone replacement therapy. Genetic counselling helps clarify the X-linked inheritance pattern, implications for future family planning, and carrier testing for female relatives. Identifying at-risk male relatives through molecular testing enables the early initiation of hormone replacement therapy, preventing life-threatening adrenal crises.

The prevention of adrenal crisis remains a cornerstone of management. Families must be educated and trained to recognise warning signs and to correctly implement stress-dose hydrocortisone protocols during illness, surgery, or trauma. The patient carries an emergency card with diagnosis details, current hydrocortisone dosing, and instructions for parenteral administration if oral medication cannot be taken. During acute episodes, glucose and sodium supplementation may also be required [41].

Replacement therapy with hydrocortisone and fludrocortisone is essential to ensure adequate electrolyte compensation and prevent adrenal crisis. In our case, hormonal control remained challenging. Hydrocortisone doses in our patient transiently exceeded the range commonly recommended for paediatric primary adrenal insufficiency because of persistently marked ACTH elevation despite adequate mineralocorticoid replacement and stable clinical condition. Although the current CAH guidelines emphasise the potential detrimental effects of prolonged supraphysiological glucocorticoid exposure on growth and metabolic outcomes, treatment in selected cases of AHC may require individualised adjustments to improve biochemical control and prevent chronic ACTH hyperstimulation. In our patient, careful longitudinal monitoring showed preserved growth velocity and bone age progression despite the temporary use of doses above 14 mg/m^2^/day. Following the switch to hydrocortisone granules (Alkindi^®^), a reduction in ACTH levels was observed. Nevertheless, it remains unclear whether this improvement was attributable to enhanced dosing accuracy, differences in absorption or formulation characteristics, or other factors such as adherence, timing of administration, or the natural course of the disease. As this observation derives from a single case, no causal inference can be made. The current evidence does not support the preferential use of one hydrocortisone formulation over another [42]. Larger controlled studies are warranted to assess the potential impact of formulation-related factors on clinical and biochemical outcomes and to investigate possible genotype–phenotype correlations.

## 5. Conclusions

This case highlights the importance of a stepwise, comprehensive diagnostic approach in suspected PAI, particularly when initial biochemical and genetic testing is inconclusive. Low 17OHP levels coupled with persistently high ACTH levels could be a red flag for AHC in male patients. Documenting novel variants not only extends the known genetic and clinical spectrum but also provides essential insights into disease pathophysiology, long-term prognosis, and reproductive outcomes, while offering crucial guidance for family screening and genetic counselling.

## Figures and Tables

**Table 1:** Laboratory data of patient at first evaluation and during follow-up. Reference ranges of our laboratory are provided in Supplementary Material. Abbreviations: N/A: not available.

AGE	10 Days	1 Months	2 Months	1 Year	3 Years	8 Years	11 Years
Plasma sodium (mEq/L)	133	133	135	135	143	137	137
Plasma potassium (mEq/L)	8.2	6.2	6.09	5	3.8	4.2	4.6
Chloride (mEq/L)	94	104	97	104	N/A	N/A	N/A
Calcium (mg/dL)	12.1	10.6	9.6	10.3	N/A	N/A	N/A
Urine creatinine (umol/L)	8.4	N/A	N/A	N/A	N/A	N/A	N/A
Urine sodium (mEq/L)	40	N/A	N/A	N/A	N/A	N/A	N/A
Urine potassium (mEq/L)	19.2	N/A	N/A	N/A	N/A	N/A	N/A
LDH (U/L)	686	N/A	N/A	N/A	N/A	N/A	N/A
Creatinkinase (U/L)	460	N/A	N/A	N/A	N/A	N/A	N/A
C reactive protein (mg/dL)	1.5	0.8	N/A	N/A	N/A	N/A	N/A
Total cholesterol (mg/dL)	N/A	N/A	N/A	106	N/A	N/A	N/A
LDL cholesterol (mg/dL)	N/A	N/A	N/A	47	N/A	N/A	N/A
HDL cholesterol (mg/dL)	N/A	N/A	N/A	32	N/A	N/A	N/A
Triglycerides (mg/dL)	N/A	N/A	N/A	53	N/A	N/A	N/A
Lactate (mmol/L)	1.3	N/A	N/A	N/A	N/A	N/A	N/A
17OHP (ng/mL)	1.6	0.8	0.6	0.6	0.06	0.12	<0.04
Androstenedione (ng/dL)	3.8	1.8	0.3	<0.3	<0.3	<0.3	<0.3
DHEAS (mcg/L)	**<150 ↓ LOW**	**<150 ↓ LOW**	**<150 ↓ LOW**	**<150 ↓ LOW**	**<150 ↓ LOW**	**<150 ↓ LOW**	**<150 ↓ LOW**
Testosterone (ng/dL)	**10.1 ↑ HIGH**	N/A	N/A	N/A	**21 ↑ HIGH**	0.04	<0.024
Cortisol (mcg/dL)	6.7	4		**<0.1 ↓ LOW**	1.4	16.3	N/A
ACTH (pg/mL)	**819 ↑ HIGH**	**622 ↑ HIGH**	**49.6**	**788 ↑ HIGH**	**>1500 ↑ HIGH**	**>1500 ↑ HIGH**	**701.7 ↑ HIGH**
Renin (mcU/mL)	**4012 ↑ HIGH**	**1360 ↑ HIGH**	N/A	**242 ↑ HIGH**	N/A	**101.6 ↑ HIGH**	**45.8**
Aldosterone (ng/dL)	5.2	4	N/A	1.2	N/A	2.6	1.4
LH (U/L)	N/A	N/A	N/A	N/A	N/A	<0.07	0.1
FSH (U/L)	N/A	N/A	N/A	N/A	N/A	0.5	0.4

**Table 2:** Clinical manifestations of AHC throughout the lifespan.

Age Group	Primary Manifestations	Details
Neonates & Infants	Adrenal crisis	Life-threatening salt-wasting crisis, often within the first weeks of life—vomiting, dehydration, hypotension, lethargy, failure to thrive
	Mineralocorticoid deficiency	Hyponatremia, hyperkalaemia, metabolic acidosis, salt craving (when older)
	Glucocorticoid deficiency	Hypoglycaemia (can cause seizures), poor stress response, hyperpigmentation (due to elevated ACTH)
Childhood	Recurrent adrenal insufficiency	Ongoing risk of adrenal crises triggered by illness, surgery, or stress if undertreated
	Growth & development	Generally normal if adequately replaced
	Hyperpigmentation	Persistent skin and mucosal darkening from chronic ACTH elevation
Puberty & Adolescence	Continued adrenal insufficiency	Lifelong glucocorticoid and mineralocorticoid dependence; stress-dose steroids required for illness or surgery
	Hypogonadotropic hypogonadism	Delayed or absent puberty—low LH/FSH, low testosterone, absent secondary sexual characteristics
	Cryptorchidism	Undescended testes (present from birth but may become clinically significant at puberty)
Adulthood	Infertility	Impaired spermatogenesis; azoospermia is common even with hormone replacement
	Continued adrenal insufficiency	Lifelong glucocorticoid and mineralocorticoid dependence; stress-dose steroids required for illness or surgery
	Psychosocial impact	Challenges related to infertility and chronic disease management

## Data Availability

Research data are available from the corresponding author on reasonable request.

## Supplementary Materials

The following supporting information can be downloaded at: https://www.mdpi.com/article/10.3390/genes17060640/s1, Table S1: Reference ranges for laboratory data.

## Abbreviations

The following abbreviations are used in this manuscript:

CAH	congenital adrenal hyperplasia
PAI	primary adrenal insufficiency
DAX1	dosage-sensitive sex reversal
AHC	adrenal hypoplasia congenita
17OHP	17α-hydroxyprogesterone
DHEAS	dehydroepiandrosterone sulphate
HH	hypogonadotropic hypogonadism
GH	growth hormone
