# Supplementary material for: NR0B1 Gene Variants as Rare Forms of Primary Adrenal Insufficiency in Children: Case Report and Narrative Review
Source: Genes (Basel). 2026 May 31;17(6):640. doi: 10.3390/genes17060640 (PMC13299720; doi:10.3390/genes17060640)
Supplement: Supplementary file 1 [file genes-17-00640-s001.zip › genes-4333359-supplementary.pdf]

genes

MDPI

Supplementary Material

Supplementary Table 1. Reference ranges for laboratory data.

<table>
<tr><th>Laboratory tests
(unit of measurement)</th><th>Age/ Tanner Stage</th><th colspan="2">Reference ranges</th></tr>
<tr><td></td><td></td><td colspan="2"></td></tr>
<tr><td rowspan="5">Sodium
(mmol/L)</td><td>Newborn (0 -30 days)</td><td colspan="2">133-146</td></tr>
<tr><td>Infant (1 month – 1 year)</td><td colspan="2">134-143</td></tr>
<tr><td>Early childhood (1 – 6 years)</td><td colspan="2">136-145</td></tr>
<tr><td>Middle childhood (6-12 years)</td><td colspan="2">137-145</td></tr>
<tr><td>Adolescence (12-18 years)</td><td colspan="2">137-145</td></tr>
<tr><td rowspan="5">Potassium
(mmol/L)</td><td>Newborn (0 -30 days)</td><td colspan="2">3.7-5.9</td></tr>
<tr><td>Infant (1 month – 1 year)</td><td colspan="2">3.7-5.5</td></tr>
<tr><td>Early childhood (1 – 6 years)</td><td colspan="2">3.6-5.4</td></tr>
<tr><td>Middle childhood (6-12 years)</td><td colspan="2">3.6-5.0</td></tr>
<tr><td>Adolescence (12-18 years)</td><td colspan="2">3.6-4.8</td></tr>
<tr><td rowspan="5">Chloride
(mmol/L)</td><td>Newborn (0 -30 days)</td><td colspan="2">96-111</td></tr>
<tr><td>Infant (1 month – 1 year)</td><td colspan="2">96-108</td></tr>
<tr><td>Early childhood (1 – 6 years)</td><td colspan="2">96-107</td></tr>
<tr><td>Middle childhood (6-12 years)</td><td colspan="2">97-107</td></tr>
<tr><td>Adolescence (12-18 years)</td><td colspan="2">97-107</td></tr>
<tr><td rowspan="5">Calcium (mmol/L)
(conversion factor from mmol/L to mg/dL for calcium is approximately 4)</td><td>Newborn (0 -30 days)</td><td colspan="2">1.90-2.60</td></tr>
<tr><td>Infant (1 month – 1 year)</td><td colspan="2">2.20-2.70</td></tr>
<tr><td>Early childhood (1 – 6 years)</td><td colspan="2">2.20-2.65</td></tr>
<tr><td>Middle childhood (6-12 years)</td><td colspan="2">2.15-2.55</td></tr>
<tr><td>Adolescence (12-18 years)</td><td colspan="2">2.10-2.55</td></tr>
<tr><td rowspan="5">Urine creatinine
(mg/dL)</td><td>Newborn (0 -30 days)</td><td colspan="2">7-17</td></tr>
<tr><td>Infant (1 month – 1 year)</td><td colspan="2">7-31</td></tr>
<tr><td>Early childhood (1 – 6 years)</td><td colspan="2">7-50</td></tr>
<tr><td>Middle childhood (6-12 years)</td><td colspan="2">14-120</td></tr>
<tr><td>Adolescence >12 years</td><td colspan="2">20-200</td></tr>
<tr><td rowspan="5">FENa (%)</td><td>Newborn (0 -30 days)</td><td colspan="2">2.5%</td></tr>
<tr><td>Infant (1 month – 1 year)</td><td colspan="2">1%</td></tr>
<tr><td>Early childhood (1 – 6 years)</td><td colspan="2">1%</td></tr>
<tr><td>Middle childhood (6-12 years)</td><td colspan="2">1%</td></tr>
<tr><td>Adolescence >12 years</td><td colspan="2">1%</td></tr>
<tr><td rowspan="5">LDH
(U/L)</td><td>Newborn (0 -30 days)</td><td colspan="2">290-775</td></tr>
<tr><td>Infant (1 month – 1 year)</td><td colspan="2">200-640</td></tr>
<tr><td>Early childhood (1 – 6 years)</td><td colspan="2">435-740</td></tr>
<tr><td>Middle childhood (6-12 years)</td><td colspan="2">370-655</td></tr>
<tr><td>Adolescence (12-18 years)</td><td colspan="2">315-570</td></tr>
<tr><td rowspan="5">CPK
(U/L)</td><td>Newborn (0 -30 days)</td><td colspan="2">40-474</td></tr>
<tr><td>Infant (1 month – 1 year)</td><td colspan="2">40-280</td></tr>
<tr><td>Early childhood (1 – 6 years)</td><td colspan="2">40-195</td></tr>
<tr><td rowspan="2">Middle childhood (6-12 years)</td><td>Male</td><td>40-195</td></tr>
<tr><td>Female</td><td>35-155</td></tr>
</table>

	Newborn (0 -30 days)		Male	45-250
			Female	30-135
Lactate (mmol/L)	Newborn (0 -30 days)		0.5-3.0	
	Infant / Childhood		0.5-2.2	
	>12 years		0.5-2.0	
Total cholesterol (mg/dL)	Acceptable		170	
	Borderline		170-199	
	High		≥200	
LDL cholesterol (mg/dL)	Acceptable		110	
	Borderline		110-129	
	High		≥130	
HDL cholesterol (mg/dL)	Low		40	
	Acceptable		>45	
	Borderline		40-45	
	High		N/A	
Triglycerides (0-9 years) (mg/dL)	Acceptable		75	
	Borderline		75-99	
	High		≥100	
Triglycerides (10-19 years) (mg/dL)	Acceptable		90	
	Borderline		90-129	
	High		≥130	
17-OHP (ng/dL)	Borderline		75-99	
	High		≥100	
	1 – 6 years		5-80	
	6 – 12 years		5-115	
	>12 years		15-200	
Androstenedione (ng/dL)	1 – 4 wk		20-290	
	1-12 months		5-50	
	1 – 6 years		5-30	
	6 – 12 years		5-50	
	>12 years		30-200	
Cortisol (mcg/dL)	1 – 4 wk		1-24	
	1-12 months		2-20	
	1 – 6 years		3-21	
	6 – 12 years		3-21	
	>12 years		5-25	
ACTH (pg/mL)	1 – 4 wk		10-185	
	1-12 months		6-48	
	1 – 6 years		6-48	
	6 – 12 years		6-48	
	>12 years		6-48	
FSH (mUI/mL)	Tanner (F) 1	< 9,2 anni	1-4.2	
	Tanner (F) 2	9,2–13,7 anni	1-10.8	
	Tanner (F) 3	10–14,4 anni	1.5-12.8	
	Tanner (F) 4	10,7–15,6 anni	1.5-11.7	

https://doi.org/10.3390/xxxxx

	Tanner (F) 5	11,8–18,6 anni	1-9.2
	Adult	Follicular phase	1.8-11.2
		Ovulatory phase	6-35
		Luteal phase	1.8-11.2
	Tanner (M) 1	< 9,8 anni	0.26-3
	Tanner (M) 2	9,8–14,5 anni	1.8-3.2
	Tanner (M) 3	10,7–15,4 anni	1.2-5.8
	Tanner (M) 4	11,8–16,2 anni	2-9.2
	Tanner (M) 5	12,8–17,3 anni	2.6-11
	Adult	2-9.2	
LH (mUI/mL)	Tanner (F) 1	< 9,2 anni	0.02-0.18
	Tanner (F) 2	9,2–13,7 anni	0.02-4.7
	Tanner (F) 3	10–14,4 anni	0.10-12
	Tanner (F) 4-5	10,7–15,6 anni	0.4-11.7
	Adult	Follicular phase	2-9
		Ovulatory phase	18-49
		Luteal phase	2-11
	Tanner (M) 1	< 9,8 anni	0.02-0.3
	Tanner (M) 2	9,8–14,5 anni	0.2-4.9
	Tanner (M) 3	10,7–15,4 anni	0.2-5
	Tanner (M) 4	11,8–16,2 anni	0.4-7
	Adult		1.5-9
Testosterone (nmol/L) TV: testicular volume (conversion factor from mmol/L to ng/dL for calcium is approximately 28.8)	Tanner (M) 1	0.5	TV: 4
	Tanner (M) 2	0.4-2.4	TV:4-8
	Tanner (M) 3	2.1-9.5	TV:8-10
	Tanner (M) 4	4.9-17.9	TV:10-20
	Tanner (M) 5	11.1-26.9	TV:20-25
Dehydroepiandrosterone sulfate (DHEA-S) (ng/dL)	Tanner (F) 1	< 9,2 anni	19–114
	Tanner (F) 2	9,2–13,7 anni	34–129
	Tanner (F) 3	10–14,4 anni	32–326

	Tanner (F) 4	10,7–15,6 anni	58–260
	Tanner (F) 5	11,8–18,6 anni	44–248
	Tanner (M) 1	< 9,8 anni	13–83
	Tanner (M) 2	9,8–14,5 anni	42–109
	Tanner (M) 3	10,7–15,4 anni	48–200
	Tanner (M) 4	11,8–16,2 anni	102–385
	Tanner (M) 5	12,8–17,3 anni	120–370
Renin (mU/L)	1 – 4 wk		31-312
	1-12 months		31-109
	1 – 6 years		23-94
	6 – 10 years		23-62
	>10 years		12-31
Aldosterone (pg/mL) The conversion factor from pg/mL to ng/dL is 0.1	1 – 4 wk		50-900
	1-12 months		50-600
	1 – 6 years		20-200
	6 – 10 years		15-150
	>10 years		15-150

https://doi.org/10.3390/xxxxx
